# Here comes the water: Risk assessments, observation and knowledge of Ompundja village

**DOI:** 10.4102/jamba.v11i1.507

**Published:** 2019-01-10

**Authors:** Loide Shaamhula, Gert van Rooy

**Affiliations:** 1 Department of Military Geography, University of Namibia, Namibia; 2 Multidisciplinary Research Centre, University of Namibia, Namibia

## Abstract

Floods in Namibia are more pronounced than drought or any other natural disaster. Ompundja village in northern central Namibia has experienced severe flooding over the last decade since the village is a catchment area of water from two distinct sources, that is, the Cuvelai system and the Efundja. Data were collected from households based on an action learning cycle. The cycle starts from context, observation, knowledge and action. A questionnaire based on 14 indicators of the action learning cycle was used to collect the needed information. Answers were recorded on a scale of 1–5, with 1 = not at all and 5 = comprehensively. In terms of the scoring, results indicate that disasters are a common phenomenon in this area. The main contributing factor is not so much of high levels of rainfall but water from the flooding basin. The flooding basin in this regard is mostly the catchment area of water from the two distinct sources, that is, Cuvelai system and the Efundja. In addition, the village also gets flooded because of the poor strategic planning and the lack of resources that would enhance fundamental changes in the livelihood of the local community. For the community to tackle disaster issues, their average score was 3.325. In terms of observation, they scored 3.667. For their involvement in risk assessments, for knowledge (traditional) and for disaster management, the score was 3.25. The same score (3.25) was observed for action and disaster mitigation as well. Based on the findings of this study, it can be concluded that communities struggle to deal with floods whenever they occur. They experience difficulties in obtaining resources as in most cases disaster is mostly viewed as a top-down approach. Communities cannot make their own decisions and in most cases traditional knowledge is discarded. Thus, it is recommended that traditional knowledge should be explored extensively in order for the community to become self-reliant.

## Introduction

Risk reduction measures are most successful when they involve the direct participation of the people most likely to be exposed to hazards. Local leaders, including both women and men, drawn from political, social and economic sectors need to assume a primary responsibility for the protection of their communities (United Nations International Strategy for Disaster Risk Reduction [UNISDR] 2011:177). Disaster reduction is most effective at the community level where specific local needs can be met. When used alone, government and institutional interventions often prove to be insufficient and frequently they only respond to crises.

Communities need to be aware of the importance of disaster reduction for their own well-being. It is important for communities to understand that ‘the whole is more important than the sum’ (Norris et al. 2007:128). Communities are capable of interpreting risks correctly, solve problems that face them. It then becomes necessary to identify and impart essential skills that can translate risk awareness into concrete practices of sustained risk management (UNISDR 2011:178). Such an approach needs activities that strengthen communities’ capacities to identify and cope with hazards and more broadly to improve residents’ livelihoods (UNISDR 2011:175).

Communities^1^ have the potential to function effectively and adapt successfully in the aftermath of disasters. As stated by Norris et al. (2007), community resilience is defined as a process linking a network of adaptive capacities (resources with dynamic attributes) to adaptation after a disturbance or adversity. Community resilience emerge from four primary sets of adaptive capacities – economic development, social capital, information and communication and community competence – that together provides a strategy for disaster readiness (Norris et al. 2007:127). Resilience is defined as (UNISDR 2009):

[…] the ability of a system, community or society exposed to hazards to resist, absorb, accommodate to and recover from the effects of a hazard in a timely and efficient manner, including through the preservation and restoration of its essential basic structures and functions. (p. 24).

To build collective resilience,^2^ communities must reduce risk and resource inequities, engage local people in mitigation, create organisational linkages and boost and protect social supports. They must have a plan which requires flexibility, decision-making skills and trusted sources of information that function in the face of the unknown (Norris et al. 2007). Moreover, from the realisation that hazards continue to distract many people’s lives and that government resources are never sufficient to assist each and every disaster victim, the Hyogo Framework for Action (2005) introduced the concept of resilience because governments have realised that they need to refocus attention away from formal reactive response measures. Instead, they need to concentrate on community resilience.

Disaster *per se* in most cases cannot be regarded as a single event but it can be regarded as something that is suffered by many communities on a continuous scale. It can be multiple and have a simultaneous effect on their lives and their community. It can, therefore, be argued that while hazards continue to destroy the lives of many people all over the world, the capacity to deal with such impacts of hazards has not been emphasised as it heavily depends on the capacity and the resources that the community might have. The point of illustration is, therefore, that in most cases rural residents suffer the impact of hazards much more when compared with urban residents. This can be explained by lack of structures in rural communities. As communities are integral in driving information upwards and downwards between the local and national levels of society, they can be used as a framework in which to develop skills and capabilities that will help people become more resilient. It is crucial to enhance resilience of communities directly affected by hazards. If a community is capacitated, it is able in terms of its social systems to organise itself to increase its capacity for learning from past disasters for better future protection and to improve risk reduction measures (Manyena 2006:437). In discussing community resilience from the point of the Ompundja village, this article will adopt the definition from Norris et al. (2007) who state that resilience can be regarded as a set of adaptive capacities that function against the continuum of adaption after a disturbance (Norris et al. 2007:130).

While Namibia is not immune to these conditions, Ompundja village in the north-central part of the country has been flooded over the past 8 years. The village is believed to be receiving high rainfall annually as well as water from the Cuvelai system. The Cuvelai system is water flowing from Angola on its way to the Etosha Pan. Water flows into the village covering the whole village. Sometimes, in many cases, water levels reach waist level and completely cut off the village from the rest of the world. School children are unable to go to school, livestock have nowhere to feed and families are unable to prepare meals as the land surface is submerged in water. As a result, the community does nothing else rather than wait for the government to help them. In most cases, helicopter crews from the Namibian Defence Force (NDF) temporarily relocate community members until the water subsides. In addition, they provide relief in the form of food. This article addresses the potential of Ompundja village to become a resilient community. What are the possibilities for the community to become self-reliant?

### Background of Ompundja village

Flooding in Ompundja can be traced back to 1952 (Respondent 2012) when such floods were seasonal, occurring at the interval of about 18 years or more. Back then the impact of these floods was minor until the early 1970s. In the 1980s, the interval of these floods decreased but their frequency increased. An increase in the frequency of the flood events in the community also lead to an increased impact. These can be associated with increased population density. In 2008, the village experienced the worst floods ever recorded in history (Wilhelm 2012). Since then, the trends of the floods changed and Ompundja village was then flooded annually. According to the assessment report by the Ministry of Agriculture, Water and Forestry (MAWF) (2012), the village was flooded annually because of some developments that were taking place in the region which diverted the natural flow of water. Moreover, the village is believed to be located within the Cuvelai Basin which covers the most part of northern Namibia.

The Cuvelai-Etosha Basin is a water catchment area located in the north-central part of Namibia. The Cuvelai-Etosha River Basin is also regarded as an interconnected system of trivial water courses, called ‘iishanas’, (Integrated Water Resource Management [IWRM] 2013). The water in the basin comes from local rainfall, runoff in ephemeral rivers – some originating from southern Angola and aquifers (underground water storage areas) (IWRM 2013). The Cuvelai drainage system originates in Angola and spreads across the flat plains of the north-central parts of Namibia, resulting in shallow ephemeral watercourses spreading throughout the area (IWRM 2013). According to the IWRM (2013), major floods (called ‘Efundja’) from local rainfall and floods from Angola contribute to the formation of a wide network of waterways (called the Cuvelai Delta). These waterways drain into the lakes within the plains and thereby cover large parts of the area in water during the rainy season. It is this water that eventually feeds into the Etosha Pan (IWRM 2013).

Because of increasing population coupled with modernisation and the general developments that have taken place since the year 2000, it has been found that development has taken place within the plains which are originally the natural waterways in the delta (Shifidi, 2014). Such developments did not only benefit the local people but also negatively impacted them as the natural flow of water has now been diverted. The natural water flow has been diverted such that it flows through the households of the village. The village has witnessed an increasing frequency of floods in the area which is attributed to the impact of climate change which is experienced all over the world and the unpredictable rainfall patterns.

During the floods, numerous houses and *Omashisha* (cereal storage) in the village are washed away. Water swamps nearby roads and homes, and many *Omahangu* storage facilities normally get submerged underwater. Water normally reaches waist-height such that it becomes difficult for residents to live in their homes. During a flooding season, the residents are unable to pound their *Omahangu* (*Pennisetum glaucum*) cereal. Livestock starve as the land surface is always submerged in water. As a result, the annual crop harvest output is diminished and general harvesting is limited because the cereals produced are unable to last until the following year. In times of floods, when someone falls ill, the only means of transport to the nearest health facility is by means of air transport, often provided by the Office of the Prime Minister (OPM).

## Methods

This study made use of a blend of quantitative tools and social science data gathering tools and presentation approaches. Interviews were conducted with heads of households of community members in the village whose houses were usually severely affected by the annual floods. A total of about 30 participants were interviewed in the village using a snowball sampling method. Interviews were conducted until no new information was emerging, a concept referred to as data saturation. A few key informant interviews were also conducted with the village headman and other three key authority leaders in the village. Quantitative data were collected for analysis and this was based on ‘Views from the Frontline’ (VFL) survey. The VFL uses an action learning curve. This curve shows the level of local monitoring and information gathering that is supported by discussions and analysis in order to develop mutual understanding and knowledge. This would in the end form the basis of self-organising and joint actions. The curve in itself continues as a further reflection on action leads to greater understanding and knowledge, shaping further planning and action. Questionnaires were administered face-to-face by research assistants that were trained in data collection methods. A standard questionnaire was used as developed by the enumerators of the Global Network of Civil Society Organisations for Disaster Reduction. The questionnaire covered the overview of everyday disasters and resilience within the affected communities. The questionnaire did not only focus on the community but also included other groups of informants, namely, the local government and the civil society. Furthermore, participants were asked to score progress over numbered options from 1 to 5 and a ‘don’t know’ against the indicators based on the following scoring system:

- 1 = Not at all
- 2 = To a limited extent
- 3 = To some extent
- 4 = Yes, but not in all cases
- 5 = Comprehensively

In addition to the 30 participates who participated in the study through focus group discussions, a total of 114 questionnaires were collected from the three sets of participants which brings the total of the study sample to 144 participants.

Data obtained through interviews were analysed using the content analysis method by creating themes from the data. The quantitative part of the data was analysed by carrying out statistical analysis and frequency distributions relating to participants’ perception of the hazard as per the VFL format of analysing quantitative data.

## Literature review

### Historical overview of disasters in Namibia

The main form of disaster that occurs in Namibia is mainly floods. Although the country has also experienced droughts and insignificant earthquakes over the past eight years, floods have been the main hazards destroying livelihoods of many. Floods have been a major problem in Namibia, affecting the majority of the population, especially those living in the north-central regions of Oshikoto, Omusati, Ohangwena and Oshana, and the north-eastern regions of Kavango and Caprivi. The floods that affect these areas vary. The floods that affect the north-central regions are basically because of high levels of rainfall received, the Efundja as well as the locality within the Cuvelai Basin. However, the floods that affect the north-eastern region are because of the floodwaters of the Zambezi River and this affects people living in low-lying areas. In addition, the floods damage infrastructure and crops, and hamper patients’ access to healthcare and children going to school. It should be pointed out that flooding mostly occurs in the Cuvelai Basin in northern Namibia, in the areas around Oshakati and result in Ompundja becoming the worst affected area.

Since 2008, Namibia has been experiencing heavy rains in the north and north-eastern parts, which resulted in severe flooding. The heavy rains were exacerbated by the rainfall received in the neighbouring countries of Angola and Zambia. The country experienced a number of hazards between 2004 and 2009 in the south, north and north-east. Between 1982 and 2008, the country had close to 19 events of natural flood disasters, which affected as much as 885 000 people. These floods caused destruction to infrastructure, homes and property; loss of agricultural production; and loss of lives. Between 2000 and 2007, the country experienced at least five crippling droughts which affected between 300 000 and 700 000 people in all the 13 regions (Kobetsu 2010).

Drawing from literature and the fieldwork conducted in northern Namibia, this article focuses on the concept of enhancing resilience among affected community members of Ompundja village.

### Main causes and patterns of vulnerability affecting Namibia’s communities

Namibia is considered to be one of the most vulnerable countries in terms of climate change in sub-Saharan Africa. The expected climate change impact includes a decline in water availability; rising average temperatures because of higher evapotranspiration; and changing rainfall patterns (Amadhila et al. 2013:2).

Because of dry land, a hot and dry climate and an erratic rainfall pattern, Namibia is exposed to recurrent droughts and wildfires. Flooding also occurs along Namibia’s international borders where perennial rivers drain into the northern plains. The northern border river systems are characterised by seasonal flooding of ephemeral drainage networks in a semi-arid environment. Major floods occurred in 1960 and in the 1970s in the Caprivi and Okavango River border zones (Amadhila et al. 2013:4). Sudden exceptional floods in 2008 and 2009, possibly as a result of increased climate variability, caused emergency disaster conditions for the local population and infrastructural development.

The areas that were worst affected were the north-central regions of Omusati, Ohangwena and Oshana and the Oshikoto and Okavango regions in the north-east of the country. These regions are mostly lying within the Cuvelai River Basin, which is shared with Angola. The Caprivi and Kavango regions in the north-east of the country were also badly affected by this flooding.

### Enhancing resilience of communities

According to Twigg (2007:7), resilience is defined as the capacity of a system to absorb recurrent disturbances, such as natural disasters, so as to retain essential structures, processes and feedbacks, and is important for many reasons. Firstly, it helps evaluate hazards holistically; secondly, it puts the emphasis on the ability of a system to deal with a hazard, absorbing the disturbance or adapting to it; and thirdly, it is forward-looking and helps explore policy options for dealing with uncertainty and future change (Berkes 2005:284). Other authors also define community resilience as the capacity of a system in a community to adapt in order to sustain an acceptable level of function, structure and identity (Edwards 2009: 2). The responsibility of a community to be resilient lies at all levels of society, local and individual resilience is central to building a resilient nation.

As illustrated by the literature, resilience is often described as a notion whereby communities can adapt and respond to a crisis. What is important is that the said community should be able to have strategies in place that can minimise their vulnerability in any event (Callaghan & Colton 2008:932).

#### How a community does become resilient?

Based on a new framework, the Disaster Resilience of Place (DROP) model by Cutter, et al. (2008:598) was designed to improve comparative assessments of disaster resilience at the local or community level. Even though the concept of community resilience has been stated by Norris et al. (2007:130) as a trajectory of functioning and disturbance, the argument of Cutter et al. (2008:599) seems to be a relevant definition of community resilience that states that a community is resilient when it has the ability in social system to respond and recover from disasters (Adger 2000:349). This also includes those inherent conditions that allow the community to absorb the impact and cope with an event, as well as post-event, adaptive processes that facilitate the ability of the social system of the community to reorganise, change and learn in response to a threat (Cutter et al. 2008:599).

Building a resilient community involves a number of players in its set-up and it is therefore of utmost importance to identify all these sub-sectors that makes up a community. Callaghan and Colton (2008:930) argue that building a resilient community is important to understand the interrelationship within a community and they suggest that communities comprise various forms of capital with ‘community capital’ as an important flow of the relationship. As various forms of capital are provided, it should be up to the community to find the right balance for resilience (Callaghan & Colton 2008: 938).

However, while numerous research efforts have assessed various dimensions of community resilience as stated by Norris et al. (2007:140), challenges remain in the development of consistent factors or standard metrics that can be used to evaluate the disaster resilient of communities.

#### Building spiral resilience to hazards

While vulnerability of a community is the pre-event, inherent characteristics or qualities of the social system of the community create the potential for harm. Vulnerability is a function of the exposure (who or what is at risk) and sensitivity of the system (the degree to which people and places can be harmed) (Adger 2006:270; Cutter 1996:531). On the contrary, spiral resilience deals with the ability of the community to sustain livelihoods in the face of the many everyday disasters which may be frequent. For the implementation of such activities, the skills should be addressed by local people even though powers outside their control often limit the changes they can make (VFL 2013:4).

According to the Views From the Front Line (VFL 2013:6), spiral resilience discusses building resilience by concentrating on action and learning – a process that draws together knowledge about the local risk context, encourages discussion to build understanding, faces up to different points of view and builds agreement on a shared course of action (VFL 2013:6). The spiral resilience model is described as a spiral resilience model, which explains resilience in the form of a straight continuum referred to as the 4R continuum or spiral resilience. The 4R continuum consists of the following:

Readiness →Response →Recovery →Rehabilitation

The process is a repeating cycle where community members build confidence through which they can work together and make a difference. Since there is so much communities can do and while there are usually underlying causes which are beyond the community’s control, such as decisions about resources, land use, agricultural development, building and other matters which are under the control of local and national government and businesses, become very crucial.

## Results

**FIGURE 1:**
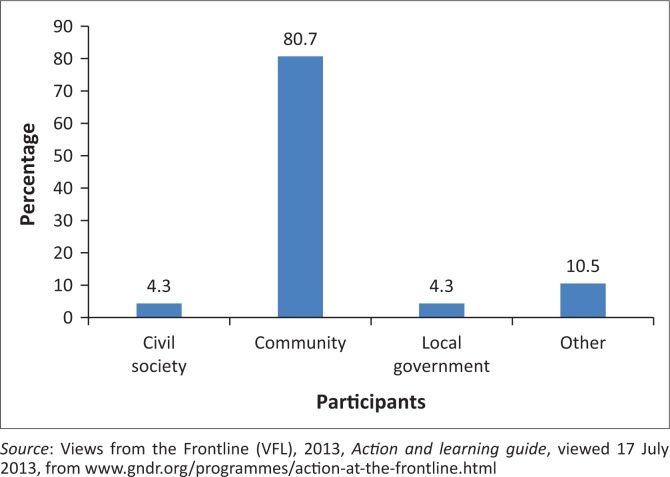
Informant groups participation.

A total of 80.7% community representatives were interviewed in comparison with local government officials (4.3%) and the civil society (4.3%) (see Figure 1). In addition, participants were generally aged between 26 and 60 years, and the majority were females constituting about 59.8% representatives of community.

### Context

The average score for the context of disasters is 3.42 (Table 1); this corresponds to ‘to some extent’. This means that to a certain extent the community tackles a number of factors that lead to disasters (floods) in the area.

**TABLE 1:** Participants views on context of risk.

Variable	Civil society	Community	Local government	Others	Average
Multi-risk resilience	3.4	3.450	3.6	4.090	3.63
Underlying causes	3.4	3.200	3.2	3.080	3.22
Average	3.4	3.325	3.4	3.585	3.42

*Source*: Adapted from Views from the Frontline (VFL), 2013, *Action and learning guide*, viewed 17 July 2013, from www.gndr.org/programmes/action-at-the-frontline.html, (mean scores)

**TABLE 2:** Participants observation and reflection of risk.

Variable	Civil society	Community	Local government	Others	Average
Risk assessment	3.600	3.480	3.4	3.500	3.5
Monitoring	4.000	3.630	4.2	3.500	3.8
Communication/public awareness	3.400	3.790	3.8	4.000	3.7
Average	3.667	3.663	3.8	3.667	3.7

*Source*: Adapted from Views from the Frontline (VFL), 2013, *Action and learning guide*, viewed 17 July 2013, from www.gndr.org/programmes/action-at-the-frontline.html, (mean scores)

**TABLE 3:** Participants views on knowledge and learning of risk.

Variable	Civil society	Community	Local government	Others	Average
Connecting	3.00	3.65	4.40	3.75	3.700
Learning	3.40	3.69	3.80	4.25	3.780
Negotiation	3.00	3.57	4.20	3.92	3.650
Conflicting resolution	3.60	3.48	3.80	3.42	3.575
Average	3.25	3.60	4.05	3.83	3.680

*Source*: Adapted from Views from the Frontline (VFL), 2013, *Action and learning guide*, viewed 17 July 2013, from www.gndr.org/programmes/action-at-the-frontline.html

### Observation and reflection

On observation and/or reflection, the average score is 3.7. This corresponds to ‘to some extent’. This implies that local community representatives to some extent are somehow involved in risk assessments, where by the local government provides information on local risk trends and regularly monitor progress to reduce such risks.

### Knowledge and learning

Regarding the knowledge and learning, the average score is 3.68. This corresponds to ‘to some extent’. This implies that to some degree the local government considers traditional knowledge when making decisions. The local leaders share risk information and local authorities together with community representatives are able to work together in decision-making and hence resolve conflicts.

### Organising and action

Lastly, ‘organising and action’ as can be seen from Table 4 consists of resources and effectiveness of early warning systems. The average score is 3.5, which corresponds to ‘to some extent’. This implies that resources are not so available to meet the needs for risk reduction. Furthermore, the average score of 3.5 also means that the local authority has a somewhat effective early warning system in place. Lastly, when the floods strike, to some extent the resources provided by the government are not sufficient to meet response needs.

**TABLE 4:** Participants views on organising and action of risk.

Variable	Civil society	Community	Local government	Others	Average
Building partnerships	3.00	3.540	4.20	3.820	3.64
Resources	3.40	3.280	3.60	2.920	3.30
Early warning	3.40	3.990	4.00	4.180	3.90
Local actions	3.00	3.280	3.60	2.830	3.11
Every disaster	3.40	3.900	3.20	3.330	3.45
Average	3.24	3.598	3.72	3.416	3.50

*Source*: Adapted from Views from the Frontline (VFL), 2013, *Action and learning guide*, viewed 17 July 2013, from www.gndr.org/programmes/action-at-the-frontline.html, (mean scores)

## Findings and discussion

While Twigg (2007:8) describes a resilient community to have capacity to recover and bounce back after an event, where capacity can be referred to as a collective way of dealing and recovering from the impact of hazards. While this capacity involves various skills by community members on how to deal with the impact of the hazard, VFL encourages a voice of addressing the underlying causes and strategies thorough discussions and interaction between local government and the community. When such information is depicted as reports which can be shared locally, with other network members, then community members have a voice in influencing the underlying causes. This is referred to as the action and learning cycle.

The concept of action and learning should be at the heart of building resilient communities as it is based on reflecting on and discussion action, which leads to new understanding, which shapes the way for further action. Therefore, the concept of action and learning is a naturally adaptive approach in responding to dynamically local conditions. Moreover, the concept of action and learning enhances social process through debate or even conflicts in search of consensus as members reflect on previous concerns and interests.

At the same time, action and learning cycle supports the development of local informal and formal governance structures, as people participate in action and learning the building of resilience, this can also be viewed as an element of driving mobilisation of the group. The concept of action and learning cycle emphasises the significance of local knowledge in building resilience. Therefore, local knowledge based on local action and learning, is regarded as a key starting point for strategy and policy for resilience and adaptation. For Ompundja community, through the concept of action and learning process, a few responses are presented below:

‘The council always informs people who are at low laying and flat areas to be evacuated to the high areas.’ (Participant 1, Female, 33 years of age)

‘We normally shop in bulk, when we know the floods are approaching, so that when we are completely cut off, we will have all we need.’ (Participant 1, Female, 33 years of age)

‘Sometimes they call us for meetings so we can share our problems but many people say apparently those meetings are for discussing the distribution of drought relief food.’ (Participant 6, Female, 46 years of age)

Further responses from Participant 1 and 6:

‘They do especially at Ombuga, they are normally helped by helicopter especially in providing food and immunisation.’

‘This does not happen anytime; it only happens once something terrible happens, for example, someone dies or perhaps the animals die. Decisions will then be made, which is not good.’

‘General comment, during floods, we experience bad situations when our animals get lost, we have to park our cars at other people’s houses, and sometimes you will find that someone has broken in your car. In addition, theft also increases during this time of flooding. We lose a lot during flooding, properties, and everything else we own.’

‘It is not easy anymore, we seek help but during flooding the help comes late as no cars are able to reach the village.’

General comment, we are pleading to our government to build more strong bridges to better the lives of people at risk.’

### On observation and reflection

Based on the findings, it appears that the community is somehow involved in the risk assessment and is able to monitor the hazards. This can be deduced from the gathering that community members hold during the time of flooding. During the floods, water surges almost covered the entire ground surface, submerging roads and washing out local routes disrupting commercial and industrial activities of the entire region. Local access to health facilities and schools is also cut off, urban sewage systems overflows with an inundation of problems associated with water supply and sewage stations. Electricity provisions also get compromised. Overall, the entire economy in the area is normally disrupted for almost 3 months as residents cannot freely move around to conduct their daily activities such as selling and buying as trade routes will be severely interrupted. The interviews reveal that, upon the realisation that water level is vastly increasing, the community leader or a village head (also known as the Settlement Disaster Risk Management Committee [SDRMCs]) would call for a community gathering where community members get to discuss the way forward. At this gathering, community members get to reflect on past experiences and how to recall the strategies that worked. It is from such gathering that community members are encouraged to apply various strategies to deal with the hazard while the community leader reports the impact of a hazard to the Constituency Counsellor for assistance. Depending on the intensity of the hazard and how it is impacting the village, the Constituency Counsellor will report through the communication channel available all the way to the OPM where assistance for hazards comes through. The local authorities indicated that there is little they can do to help those that are affected, as they are normally taken care of by the emergency unit in the OPM.

**FIGURE 2:**
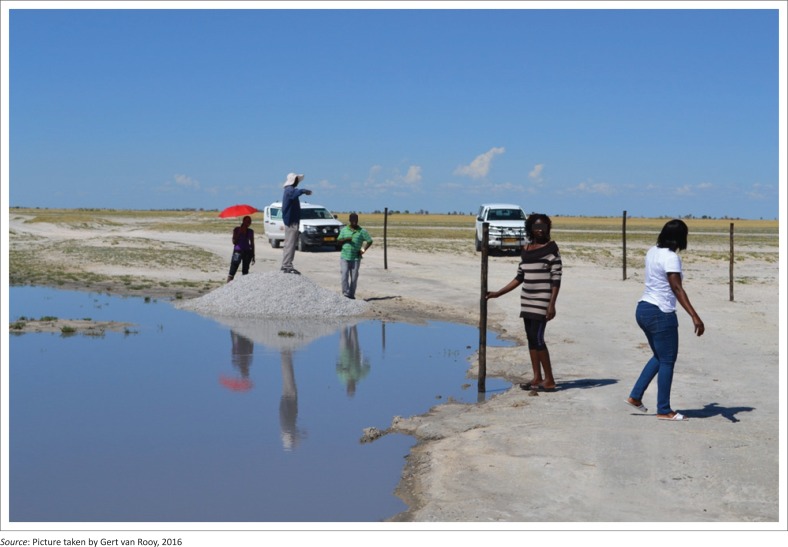
Picture at Ompundja village – The purpose of the poles is to indicate the water level to motorist and as guidance of the road to the drivers (traditional knowledge).

It is from such a gathering that community members are involved in adaptive activities. One particular activity can be seen in Figure 2, where community members erect poles on the road as a way of marking out a driveway which will direct drivers when the surface is covered in water.

According to the Namibian National Disaster Risk Management Policy, the relevant SDRMCs should assess the magnitude of the significant event or threat thereof and make recommendations to the regional governor as to whether regional or local flood disaster exists or not (Oshana Contingency Plan, n.d.:14).

The regional governor shall then announce a regional or local flood disaster in a statement made to the full council (Oshana Contingency Plan, n.d.:14).

After such an announcement, flood emergency assessments are carried out in order to measure the extent to which the livelihoods of the community have been impacted on. A rapid impact assessment is conducted on the flood situation within the affected community and a report is compiled and submitted to the RDRMC (The RDRMC coordinate disaster risk management among sector governmental institutions, local authorities, communities and other role-players involved in disaster risk management at regional level) (Disaster Risk Management Act, 2012 [Act No. 10 of 2012]) (Oshana Contingency Plan, n.d.:14). The findings of the assessments assist various sectors in redesigning their disaster risk management activities and thereby inform the response team.

The above description reveals that the community members of Ompundja village are participating in communication and public awareness activities. This also implies that the community has observed the hazards over time and that they are able to reflect on those events. In terms of observation, this is viewed as a capacity and an enormous potential to deal with the risk at the community level.

These correspond very well with the concept of observation and reflection as framed within the thinking of Freire (1996) on action and reflection as a means of empowerment. When affected communities are able to observe and reflect on past events, it is taken as a starting point in assessing the risks. When risk is properly assessed, it enables the community to determine what action can be taken to address them. In a way, the aspect of observation and reflection complements the concept of Vulnerability and Capacity Assessments (VCA) at community level (Van Aalst, Cannon & Burton 2008). Vulnerability and Capacity Assessment uses various participatory tools to gauge people’s exposure and capacity to resist natural hazards. Vulnerability and Capacity Assessment is carried out at the level of villages and urban neighbourhoods, and uses community member’s participation to diagnose vulnerabilities, assess a community’s risk priorities and work together with the people to devise ways of increasing their capacities to resist the impact of hazards (Van Aalst et al. 2008). A very good example was stated by one of the participants:

‘The headman invites the community so that they make decisions collectively.’ (Participant 8, Male, 48 year of age)

Vulnerability and Capacity Assessment enables local priorities to be identified and appropriate action to be taken to reduce disaster risk and assist in the design and development of programmes that are mutually supportive and responsive to the needs of the people most closely concerned. However, at all means the results of risk assessment need to be verified and updated regularly to ensure validity.

However, in Ompundja village, this knowledge and expertise is still untapped. There is still a need for personnel who are qualified in the concept of VCA. This is necessary as there is a need for an expert who can facilitate the development of the risk assessment skills at the village level. Therefore, it is recommended that the local government or civil society get involved at the community level and impart the knowledge of assessing risks. This is necessary since the community has the potential, but they still need experienced personnel who are able to guide and formulate priorities.

Finally, regarding the ‘organisation and action’, the community of Ompundja seems to be at an early stage of organising itself in order to respond to hazards. From the findings of this survey, it is clear that the community needs to invest in building partnerships as currently its only partner is the local government. Different organisations, sectors and disciplines need to work together to build safety and resilience in this community. Private organisations and other institutions can assist the government and the community to raise awareness, enhance education and build capacity for disaster risk reduction. Access to information for community and multi-stakeholders’ forum is an absolute need, and should be considered.

This, however, can only be achieved if the community builds partnerships with other institutions. Literature has revealed that the active participation and involvement of civil society organisations, working closely with local authorities and the affected people, is central to addressing the challenging task of building resilience of nations and communities to disasters.

‘The government officials normally come here each and every flooding time and do their statistics and the next thing we will see is flood relief food. They do not necessary give us any feedback.’ (Participant 4, Female, 35 years of age)

## Recommendations and conclusion

The vulnerability of communities in Ompundja community is increasing, more especially since that community lies within the poor rural communities living within the Cuvelai flood basin. Floods in Ompundja village are difficult to monitor because they are determined by local factors such as precipitation, gradient of the terrain, drainage of the rivers, protection devices in place, and in this case the rainfall patterns experienced over the entire Southern African region for that particular year.

Although the main contributors of recurring floods in the village can be associated with high rainfall and the fact that the community is believed to be located within a floodplain, another contributing factor could be poor strategic planning, implementation methods and lack of resources necessary to bring about practical change to the community. Therefore, the scoring of the participants reflects a degree of frequency of the hazards in the community and thereby highlights the importance of why this community needs to adapt to these changing climatic conditions as well as develop capacity to become a resilient community. The community applies numerous mechanisms to cope with floods, depending on the intensity and the impact of the hazard on their livelihoods. This study shows the potential and the capabilities the community has towards becoming a resilient community. However, there is still untapped knowledge within the community that still requires guidance and assistance from external experts.

Usually, the poor and the elderly are the victims of disaster areas; therefore, building a resilient community should consider these more vulnerable groups in the society.

The early warning systems that usually warn the community at some instances are not necessarily reliable. An example can be taken from one announcement which was received through the radio and newspaper ‘this year floods will be the worst ever, floods which were never experienced in Namibia, but what happened afterwards was the opposite’ said one participant in our group discussion. Therefore, communities have developed opinion that most scientific predictions are not so reliable and one should not always believe in them. Be that as it may, the following recommendations are befitting:

- The collaboration between the government and the traditional authorities should be strengthened in order to ensure some mutual and beneficial dependence that helps the victims. This is in view of the observations of one participant who averred that ‘our views are mostly not considered, and some offices consider us useless’.
- There is the need for a pre-needs assessment. This comes in the wake of the fact that the needs of the victims are not always necessarily the same, when floods strike, the government rushes in and ‘gives us food aid, but they do not even make some surveys to find out what we really, need, may be food is not what we need?’ as stated by a participant.
- Usually, the poor and the elderly are the victims of disaster area; therefore, building a resilient community should consider those more vulnerable groups in the society.
- The Ompundja experience will be an example of how to obtain insight on how to build capacity in protecting the community’s livelihoods and for them to become resilient to disasters.

## Footnotes

1 Communities are composed of built, natural, social and economic environments that influence one another in complex ways (Norris et al. 2007, p. 128).

2 Resilience = metaphor = return to the equilibrium after displacement.
